# Reducing Photo-Oxidative Stress in IVF: A Retrospective Analysis of Cycles with Poor Blastocyst Development

**DOI:** 10.3390/jcm15020881

**Published:** 2026-01-21

**Authors:** Krisztina Gödöny, Ákos Várnagy, Péter Mauchart, Bernadett Nagy, Kálmán Kovács, József Bódis

**Affiliations:** 1 National Laboratory on Human Reproduction, University of Pécs, H-7624 Pécs, Hungary; krisztina.godony@cdoki.hu (K.G.); varnagy.akos@pte.hu (Á.V.); bernadett.nagy@aok.pte.hu (B.N.); kovacs.kalman@pte.hu (K.K.); bodis.jozsef@pte.hu (J.B.); 2 Department of Obstetrics and Gynecology, Medical School, University of Pécs, H-7624 Pécs, Hungary; 3 Doctoral School of Health Sciences, Faculty of Health Sciences, University of Pécs, H-7621 Pécs, Hungary

**Keywords:** oxidative stress, embryo culture conditions, light exposure, blastocyst development

## Abstract

**Background:** The success of in vitro fertilization (IVF) is influenced by multiple patient- and laboratory-related factors, including maternal age, body mass index (BMI), ovarian stimulation, and embryo quality. Laboratory illumination may induce photo-oxidative stress, potentially impairing embryo development and implantation. This study evaluated the clinical impact of introducing a light-protection protocol in an IVF laboratory. **Methods:** We retrospectively analyzed 2125 IVF cycles with fresh embryo transfer performed at the Assisted Reproduction Centre of the University of Pécs between 1 March 2016 and 30 November 2020. A light-protection protocol was implemented on 1 March 2017, while all other laboratory and clinical parameters remained unchanged. Pregnancy outcomes before and after implementation were compared, with additional subgroup analyses focusing on cycles with low blastocyst-formation rates. **Results:** After implementation of light protection, overall pregnancy rates increased by approximately 5%; however, this difference was not statistically significant. In contrast, subgroup analyses demonstrated a markedly greater improvement in pregnancy outcomes—up to 37%—in cycles characterized by low blastocyst-formation rates. **Conclusions:** Although light protection did not significantly improve overall pregnancy rates, the findings suggest a clinically relevant benefit in selected cases with reduced embryonic developmental competence. Minimizing photo-oxidative stress may therefore represent a targeted laboratory intervention to improve IVF outcomes in vulnerable embryo populations.

## 1. Introduction

In vitro fertilization (IVF) has brought about a revolutionary breakthrough for couples struggling with infertility, offering an effective solution when natural conception is impaired. The success of IVF depends not only on patients’ biological characteristics—such as maternal age, body mass index, and the effectiveness of hormonal stimulation [1,2]—but also on the optimization of the laboratory environment [3]. Embryo quality, often assessed by the proportion of embryos reaching the blastocyst stage, plays a crucial role in implantation and the establishment of a clinical pregnancy [4].

In recent years, increasing attention has been directed toward the extent to which laboratory illumination contributes to the development of oxidative stress, potentially harming developing embryos [5,6]. Our previous work demonstrated that reducing light-induced toxic effects—particularly through spectrally modified, low-intensity illumination, red filters, aluminum shielding, and UV/IR blocking—may offer substantial benefits in preserving embryo viability and quality [7,8]. The aim of our development was to minimize the molecular damage induced by light exposure, thereby reducing embryo loss during gamete manipulation, fertilization, and embryo culture [7].

Despite increasing experimental and clinical interest in oxidative stress as a modifiable factor in assisted reproduction, its clinical relevance remains incompletely defined. Oxidative stress has been implicated in impaired oocyte and embryo development; however, published studies report heterogeneous and sometimes contradictory associations with IVF outcomes, depending on laboratory conditions, patient characteristics, and outcome measures used [9].

With regard to light exposure specifically, experimental and preclinical studies consistently demonstrate phototoxic effects on gametes and embryos, yet systematic clinical evidence remains limited, and the extent to which light-protection strategies translate into measurable improvements in clinical outcomes is still insufficiently explored [10].

Although light exposure is an inherent and unavoidable component of IVF laboratory procedures, its clinical relevance remains insufficiently defined. It is unclear whether reducing light-induced stress provides uniform benefits across all treatment cycles, or whether its impact becomes particularly pronounced in settings where embryo developmental capacity is already compromised. Addressing this question is essential to determine the conditions under which light-mitigation protocols translate into measurable clinical improvement rather than representing a merely theoretical laboratory refinement.

The present study uses retrospective data to examine the extent to which light protection influences IVF outcomes, and how this effect may be modified by embryo quality, with special attention to cases characterized by low blastocyst development rates.

## 2. Materials and Methods

### 2.1. Data Collection

Data were collected at the Assisted Reproduction Centre of the Department of Obstetrics and Gynecology, University of Pécs, where a total of 2125 IVF cycles with fresh embryo transfer performed between 1 March 2016 and 30 November 2020 were included in the analysis. Based on our previous work, a light-protection protocol was introduced on 1 March 2017, while all other laboratory conditions—including stimulation protocols, incubator types, culture media, and incubator gas composition—remained unchanged throughout the study period, and no relevant changes in clinical stimulation practices were implemented. Consequently, two time periods were distinguished: one in which light protection was applied (after 1 March 2017), and an earlier period in which standard illumination conditions were used (1 March 2016–28 February 2017). All further methodological details, the exact description of the light-protection protocol, and the baseline clinical and laboratory characteristics of the patients are available in our previous publication [7,8]. All IVF cycles with fresh embryo transfer performed during the study period were eligible for inclusion. Cycles involving frozen embryo transfer, donor oocytes, preimplantation genetic testing, or incomplete clinical data were excluded from the analysis.

### 2.2. Statistical Analysis

The primary exposure variable of interest was the application of laboratory light protection. Maternal age, body mass index (BMI), and the FSH dose used for ovarian stimulation were recorded. Because the range of FSH doses differed markedly from that of the other variables, FSH dosage was log-transformed (lnFSH) prior to inclusion in the model to ensure appropriate scaling and comparability across predictors.

Embryo quality was assessed by the proportion of embryos reaching the blastocyst stage, treated as a continuous variable ranging from 0 to 1. Blastocyst development rate was calculated exclusively for day-5 embryo transfers, and all analyses involving this variable—including interaction models and subgroup evaluations—were restricted to day-5 transfers.

The main outcome variable was the occurrence of pregnancy, coded as a binary variable (0 = not pregnant, 1 = pregnant). Additionally, survival analysis was performed to evaluate the number of IVF cycles required to achieve pregnancy, accounting for censoring when pregnancy did not occur by the end of the study period. Kaplan–Meier curves were constructed to visualize the distribution of cycle numbers until pregnancy in the two groups, and differences between curves were tested using the log-rank test. Given that pregnancy represents a time-to-event outcome measured across successive IVF cycles, survival analysis was deemed appropriate to model the probability of achieving pregnancy over time.

Age-related trends in IVF success were visualized using LOESS-smoothed curves plotted separately for cycles performed with and without light protection. Individual data points represent the raw observations.

Subsequently, Cox proportional hazards models were constructed. First, a univariable model was built including only the light protection variable, followed by a multivariable model adjusting for maternal age, body mass index (BMI), and lnFSH to account for potential confounders. These covariates were selected a priori based on established clinical relevance and previous evidence indicating their association with IVF outcomes, and were included to account for patient-related and stimulation-related confounding factors known to influence pregnancy probability.

Particular attention was given to how the effect of light protection varied according to embryo quality. Therefore, an interaction model including light protection and blastocyst development rate was also fitted. Hazard ratios (HRs) were calculated to describe how a one-unit change in each predictor influenced the relative probability of achieving pregnancy.

For further analysis, patients were stratified into three categories based on blastulation rate: low (<33%), medium (33–66%), and high (>66%) embryo-quality groups. Within these subgroups, additional Kaplan–Meier analyses and Cox regression models were performed. All statistical analyses were conducted using R version 4.4.2 [11], with statistical significance set at *p* < 0.05.

## 3. Results

### 3.1. Cox Regression Model Outcomes

**Figure 1:**
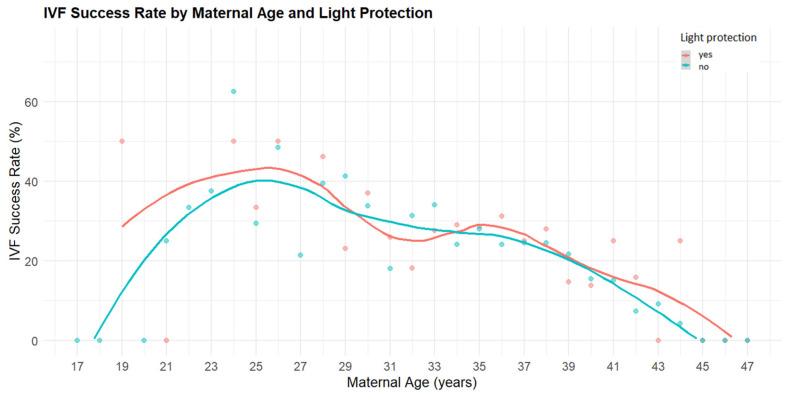
Age-dependent IVF success rates in cycles with and without light protection. The figure shows clinical pregnancy rates (%) plotted against maternal age. Each point represents an observed success rate for a given age. The red points and LOESS-smoothed red curve correspond to IVF cycles performed with light protection, while the blue points and curve represent cycles without light protection. Overall, cycles involving light protection tended to yield slightly higher pregnancy rates across most age groups, with the difference becoming more apparent in patients over 35 years of age.

In the univariable Cox model, the application of light protection was associated with a 5% increase in the probability of pregnancy; however, this effect did not reach statistical significance (Figure 1, Table 1).

In the multivariable model, which was adjusted for maternal age, body mass index (BMI), and the administered FSH dose during stimulation, light protection again showed no significant effect on pregnancy outcomes. Maternal age emerged as a strong negative predictor: each additional year was associated with an 8.46% relative reduction in the probability of achieving pregnancy (Figure 1). The log-transformed FSH dose also demonstrated a significant negative effect, with each log-unit increase reducing the chance of pregnancy by 38.13%. BMI did not significantly influence IVF success.

In the interaction model, the effect of light protection became statistically significant, particularly as a function of embryo quality. In the light-protected group, the probability of achieving pregnancy was 32% higher compared to cycles performed without light protection, and the blastocyst rate was a strong positive predictor. The interaction term between light protection and blastocyst rate was significant, indicating that light protection was most beneficial in cycles with low blastocyst formation and less beneficial in cycles with higher blastocyst rates (Table 1).

### 3.2. Subgroup Analysis (Cox Regression)

**Figure 2:**
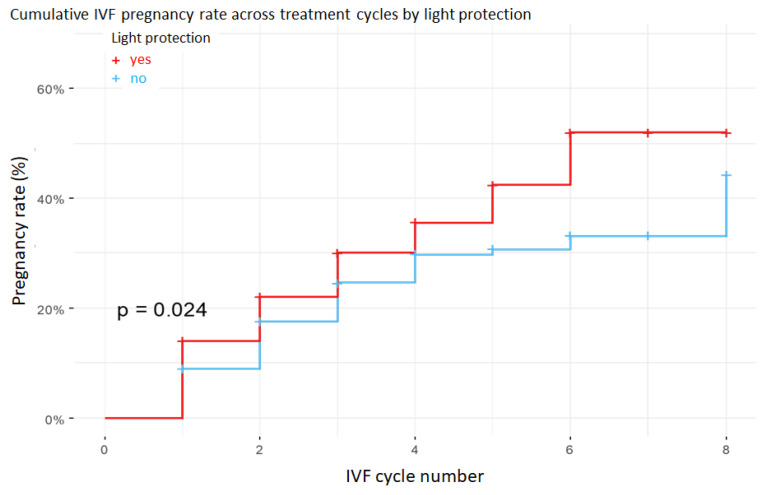
Kaplan–Meier curves showing the probability of pregnancy across successive IVF cycles in the low blastulation rate group (less than 33%). Embryos cultured under light protection (red curve) had significantly higher cumulative pregnancy probabilities than those cultured under standard illumination (blue curve), as supported by the log-rank test.

In the subgroup with low blastocyst rate (<33%), the application of light protection increased the likelihood of pregnancy by 37% compared with cycles performed without light protection, as illustrated by the subgroup-specific Kaplan–Meier curve (Figure 2) and summarized in Table 2. This suggests that in cases of poorer embryo quality, light protection may play an important role in preserving developmental potential. In the medium (33–66%) and high (>66%) blastocyst-rate groups, no statistically significant differences were observed between protected and non-protected cycles.

## 4. Discussion

The aim of the present study was to explore in detail the extent to which the application of a laboratory light-protection protocol influences IVF outcomes, with particular emphasis on embryo quality as reflected by the blastocyst-formation rate. Our findings reinforce our previous research [7,8], indicating that reducing oxidative stress can markedly improve embryo viability, especially in patients with low blastulation rates.

Although, in the overall population, the introduction of light protection resulted in only a modest ~5% improvement in pregnancy rates—an effect that did not reach statistical significance—the interaction model and the subgroup analyses clearly demonstrate that in cycles characterized by a low blastocyst development rate, targeted light-protection measures can yield substantial benefits, with improvements of up to 37%. This suggests that light-protection protocols play a critical role in mitigating oxidative stress, thereby offering significant clinical advantage particularly in cases of poorer embryo quality [10].

From a clinical perspective, it is important to emphasize that although relative measures indicated a significant benefit of light protection in low blastocyst-rate cycles, these effects also translated into meaningful absolute differences in pregnancy rates. In this subgroup, the observed improvement represents a clinically relevant gain for patients with limited embryo developmental potential, where even modest absolute increases may substantially affect treatment prognosis.

Historically, light exposure during routine embryology procedures has been considered an insignificant component of laboratory workflow. However, growing evidence suggests that visible and near-UV wavelengths can trigger photochemical reactions, generate reactive oxygen species (ROS), and induce mitochondrial and DNA damage in oocytes and pre-implantation embryos [12]. In this context, light protection refers to strategies designed to reduce cumulative light dose—such as filtered illumination, dish shielding, and workflow modifications that limit exposure during critical developmental windows [6,7]. Recent clinical findings demonstrate that reduced photo-oxidative stress is associated with improved blastocyst development and embryo quality, underscoring the translational relevance of these measures [8]. Although the procedural adjustments appear subtle, their biological impact may be substantial, particularly in embryos with inherently reduced developmental potential, where even minor environmental stress may precipitate developmental arrest [10]. Therefore, light protection should not be considered a mere technical refinement, but rather a microenvironmental optimization strategy aimed at preventing avoidable laboratory-induced embryotoxicity.

Maternal age remained the strongest negative predictor of pregnancy [13]. Importantly, its impact is not a fixed annual decrement, but a relative, percentage-based reduction that follows an exponential pattern. In practical terms, this means that with each additional year, the probability of achieving pregnancy decreases to 91.54% of the previous year’s likelihood. As a consequence, age exerts a more pronounced effect in younger patients—where baseline success rates are higher—while in older patients, who begin from a lower baseline probability, the same relative reduction has a smaller absolute effect [14]. We also observed that higher FSH doses were associated with reduced pregnancy probability, consistent with clinical experience showing that patients requiring higher doses typically exhibit weaker ovarian response and poorer prognosis [15].

From a mechanistic perspective, experimental evidence supports several biological pathways through which light exposure may impair embryo development. Visible and short-wavelength light has been shown to induce oxidative stress in oocytes and pre-implantation embryos, leading to DNA damage and impaired embryonic development in experimental models [5]. In addition, light-induced oxidative stress has been associated with mitochondrial dysfunction and reduced ATP production, potentially compromising embryonic developmental competence [16]. These mechanisms may be particularly relevant in embryos with limited developmental reserve, providing a biological explanation for the pronounced benefit of light protection observed in low blastocyst-rate cycles.

While live birth rate is widely regarded as the most clinically meaningful endpoint in assisted reproduction [17], clinical pregnancy rate may more directly reflect the impact of laboratory-level interventions. Pregnancy establishment represents an early and relatively proximal outcome of embryo quality and implantation competence, whereas progression to live birth is influenced by a wide range of maternal, obstetric, and placental factors that extend beyond laboratory control. Accordingly, laboratory-related modifications—such as optimization of the embryo culture environment—are expected to exert their primary effects at the level of implantation and early pregnancy rather than on downstream obstetric outcomes [18].

The limitations of our study include its retrospective design, which cannot fully eliminate selection bias or the influence of unmeasured confounders, as well as the non-randomized nature of the comparison groups [8]. Future prospective and randomized studies, along with biomarker-based analyses, may provide deeper insight into the cellular mechanisms of light-induced damage and further inform the development of optimized IVF laboratory protocols.

In summary, although light protection yielded only a minimal and statistically non-significant improvement in pregnancy rates in the overall cohort, targeted light-protection strategies—particularly in cases with low blastocyst-formation rates and poorer embryo quality—may confer substantial benefit. These findings not only support our earlier observations but also highlight the potentially critical role of light-protection protocols in improving clinical IVF outcomes.

## 5. Conclusions

Overall, our study demonstrates that although the implementation of a light-protection protocol yielded only a modest, non-significant ~5% improvement in pregnancy rates in the overall population, targeted light-protection measures can provide substantial benefit in specific subgroups. In cases characterized by low blastocyst-formation rates, light protection was associated with a marked improvement of up to 37% in the likelihood of achieving pregnancy. These findings underscore the importance of light-protection protocols in optimizing the IVF laboratory environment and maximizing embryo safety, particularly for patients with lower embryo quality.

## Figures and Tables

**Table 1:** Cox regression model results evaluating the relationship between light protection and pregnancy outcome.

Model	Variable	HR	95% CI	*p*-Value
Univariable model	Light protection	1.051	0.8601–1.284	0.628
Multivariable model	Light protection	0.9437	0.7626–1.1679	0.558
	Maternal age (years)	0.9154	0.8996–0.9316	<0.001 *
	BMI	1.0079	0.9861–1.0302	0.472
	FSH dose (lnFSH)	0.6187	0.5104–0.7501	<0.001 *
Interaction model	Light protection	1.3202	1.0089–1.7275	0.042 *
	Blastocyst development rate	2.9046	2.2855–3.6914	<0.001 *
	Light protection × Blastocyst development rate	0.5718	0.3487–0.9377	0.026 *

Model = type of statistical model applied; Variable = predictor included in the model; HR = Hazard Ratio; 95% CI = 95% Confidence Interval; *p*-value = statistical significance; * *p* < 0.05.

**Table 2:** Subgroup analysis of the effect of light protection across blastocyst-rate categories.

Blastocyst Development Rate Subgroup	HR	95% CI	*p*-Value
Low (<33%)	1.37	1.038–1.814	0.024 *
Medium (33–66%)	0.78	0.4713–1.279	0.320
High (>66%)	0.91	0.6338–1.305	0.607

Blastocyst development rate subgroup refers to categories based on the proportion of embryos reaching the blastocyst stage; HR = Hazard Ratio; 95% CI = 95% Confidence Interval; *p*-value = statistical significance; * *p* < 0.05.

## Data Availability

The data that support the findings of this study are available from the corresponding author upon reasonable request.
